# Ameliorative effect and mechanism of Yi‐Suan‐Cha against hyperuricemia in rats

**DOI:** 10.1002/jcla.23859

**Published:** 2021-07-12

**Authors:** Yuanyuan Qin, Xuan Zhang, Hui Tao, Yangyang Wu, Jie Yan, Lin Liao, Jianjun Meng, Faquan Lin

**Affiliations:** ^1^ Department of Clinical Laboratory The First Affiliated Hospital of Guangxi Medical University Nanning China; ^2^ Guangxi Medical College Nanning China; ^3^ The First Affiliated Hospital of Guangxi Medical University Nanning China

**Keywords:** hyperuricemia, transporter protein, uric acid, Yi‐Suan‐Cha

## Abstract

**Background:**

This study aimed to evaluate the urate‐lowering effects of Yi‐Suan‐Cha and explore its underlying mechanisms in experimental hyperuricemia induced in rats.

**Methods:**

Forty‐eight male SD rats were randomly allocated into normal control, model, allopurinol, benzbromarone, low‐dose Yi‐Suan‐Cha (0.2 g/ml), and high‐dose Yi‐Suan‐Cha (0.4 g/ml) groups (*n* = 8 rats per group). Rat models of hyperuricemia were established through intragastric administration of adenine 25 mg/kg + potassium oxalate 300 mg/kg for 3 weeks. After the last administration, serum uric acid, creatinine, and urea nitrogen levels were measured. Renal histopathology was observed by hematoxylin‐eosin staining. Xanthine oxidase level in serum and liver homogenates was measured by ELISA. The protein and mRNA expression of URAT1, ABCG2, OAT1, and GLUT9 in the kidney was detected by Western blotting and RT‐PCR, respectively.

**Results:**

The serum uric acid levels were significantly lowered in all medication groups than in the model group. The benzbromarone and both Yi‐Suan‐Cha groups showed clear kidney structures with no obvious abnormalities. Compared with the normal control group, the model group showed increased URAT1/GLUT9 protein expression and decreased ABCG2/OAT1 protein expression. Compared with the model group, both Yi‐Suan‐Cha groups showed decreased URAT1/GLUT9 protein expression and increased ABCG2/OAT1 protein expression. Compared with that in the normal control group, URAT1/GLUT9 mRNA expression increased in the model group. Compared with the model group, the low‐dose and high‐dose Yi‐Suan‐Cha groups showed decreased URAT1/GLUT9 mRNA expression and increased ABCG2/OAT1 mRNA expression.

**Conclusion:**

Yi‐Suan‐Cha may lower uric acid level by downregulating URAT1/GLUT9 expression and upregulating ABCG2/OAT1 expression.

## Graphical abstract

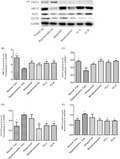

## 1 BACKGROUND

With the rapid development of economies, human lifestyles and diseases have changed significantly. One of these conditions, hyperuricemia shows increasing incidence each year in various regions of the world.^1^, ^2^, ^3^ Epidemiologic studies revealed that hyperuricemia prevalence in the rural Northeast Chinese population was 15.0% and 7.3% in men and women, respectively; especially, in the Mongolian area, the prevalence was as high as 17.7% in men.^4^, ^5^ One survey^6^ estimated hyperuricemia prevalence to be 13.19% in the coastal area of Shandong Province, China. A recent cross‐sectional study^7^ confirmed an independent correlation between hyperuricemia and reduced glomerular filtration rate in patients with chronic kidney disease (CKD). Hyperuricemia prevalence in patients diagnosed with stage 1–3 CKD is between 40% and 60% and that in patients with stage 4 or 5 CKD is 70%.^8^ Hyperuricemia is not only caused by gout but also related to obesity, hyperlipidemia, hypertension, cardiovascular and cerebrovascular diseases, diabetes, and kidney injury.^9^, ^10^, ^11^ Brodov et al^12^ reported that for every 1 mg/dl (59.5 μmol/L) increase in blood uric acid level, the risk of cardiovascular disease increases 1.45 times. Otomo^13^ retrospectively analyzed the correlation between serum uric acid level and renal injury in 81,770 hospitalized patients and found that long‐term hyperuricemia is likely to cause a decline in renal function. Hence, prevention and treatment of hyperuricemia have become global endeavors.

Currently, allopurinol is the first‐choice drug for lowering uric acid levels. However, only approximately 40% of patients treated with allopurinol achieved the therapeutic goal of serum uric acid level lower than 6 mg/dl.^14^ Febuxostat, pegloticase, probenecid, and benzbromarone are prescribed for patients who cannot tolerate allopurinol. However, hyperuricemia treatment requires long‐term medication, and drug withdrawal may induce relapse. Long‐term treatment with xanthine oxidase (XOD) inhibitors, such as allopurinol, increases the risk of adverse effects, such as headache, fever, vomiting, renal and hepatic toxicity, myelosuppression, Stevens‐Johnson syndrome, and toxic epidermal necrolysis.^15^, ^16^ Therefore, development of natural medicinal plants for treating gout and hyperuricemia with minor side effects is urgently needed.

Recently, several studies, considering the high prevalence of hyperuricemia^17^ and safety of natural products, have sought new agents from natural products for the treatment of hyperuricemia and gout.^18^ For example, traditional Chinese medicine is frequently utilized to prevent hyperuricemia‐related disorders.^18^ Hu et al^19^ found that Simiao pills promote uric acid excretion and protect the kidneys of uric acid model mice by adjusting the level of uric acid transporter protein. Moreover, Qin et al^20^ suggested that the mangiferin aglycon derivative J99745 exerts urate‐lowering effect by inhibiting XOD activity and urate transporter 1 (URAT1) expression.

Yi‐Suan‐Cha is a traditional Chinese medicine formula comprised of Chinese hawthorn leaf, Astragali Radix, and Flos Sophorae. Chinese hawthorn leaf and Astragali Radix are Chinese herbs commonly used to lower blood lipids, which may be beneficial because hyperuricemia patients often suffer from hyperlipidemia.^21^, ^22^ Thus, this study aimed to evaluate the urate‐lowering effects of Yi‐Suan‐Cha and explore its underlying mechanisms in rats induced with hyperuricemia.

## 2 METHODS

### 2.1 Galenical preparation of Yi‐Suan‐Cha

Chinese hawthorn leaf, Astragali Radix, and Flos Sophorae were purchased in a pharmacy. The crude herbs were extracted in water and filtered according to the ratio of 2:1:1 to prepare Yi‐Suan‐Cha. The resulting extract was diluted with water to prepare high‐dose (0.4 g/ml) and low‐dose (0.2 g/ml) preparations.

### 2.2 Reagents and drugs

Allopurinol was purchased from Shimao Tianjie Pharmaceutical (Jiangsu) Co., Ltd. Benzbromarone was obtained from Kunshan Longdeng Ruidi Pharmaceutical Co., Ltd. Before use, allopurinol, and benzbromarone were triturated and dissolved in distilled water to a concentration of 2 g/L. Carboxymethylcellulose, adenine, and oxonic acid potassium salt were obtained from Beijing Soleib Biotechnology Co., Ltd. Adenine and oxonic acid potassium salt were dissolved together in 0.5% carboxymethylcellulose to final concentrations of 5 g/L and 60 g/L for adenine and oxonic acid potassium salt, respectively.

### 2.3 Animal model

Forty‐eight male Sprague‐Dawley rats weighing 180–230 g (Laboratory Animal Center, Guangxi Medical University, China) were maintained under 12‐h dark/light cycle at a room temperature of 22 ± 2°C, with free access to standard chow and water. After 1 week of acclimatization to the study environment, the rats were randomly divided into normal control, model, allopurinol, benzbromarone, low‐dose Yi‐Suan‐Cha, and high‐dose Yi‐Suan‐Cha groups (*n* = 8 per group). To establish hyperuricemia models, rats in all groups, except for the normal control group, were intragastrically administered adenine (25 mg/kg) and potassium oxonate (PO) (300 mg/kg); these treatments were dissolved in 0.5% sodium carboxymethylcellulose and administered once per day for three successive weeks. At the same time, rats in the normal control group received equal volumes of 0.5% sodium carboxymethylcellulose. Next, the rats in the allopurinol, benzbromarone, low‐dose Yi‐Suan‐Cha, and high‐dose Yi‐Suan‐Cha groups were intragastrically administered 20 mg/kg allopurinol (20 mg/kg), benzbromarone (10 mg/kg), or Yi‐Suan‐Cha at 4 or 2 mg/kg, respectively; these treatments were dissolved in distilled water and administered every afternoon during hyperuricemia induction. Meanwhile, rats in the normal control and model groups received equal doses of distilled water.

### 2.4 Blood and tissues collection

Approximately 2.0 ml of blood was collected from the posterior orbital venous plexus of the rats before the experiment (before administration) and after the third week of the experiment (after the last administration). After natural coagulation was completed at room temperature, the serum was separated by centrifugation at 4000 rmp for 2 times. Simultaneously, liver tissues were excised and stored at −80°C until XOD analysis. Kidney tissues were quickly and carefully dissected on an ice plate; a part of the tissue was immediately fixed for hematoxylin and eosin (H&E) staining, whereas the other parts were stored at −80°C for PCR and Western blotting analysis.

### 2.5 Determination of levels of serum uric acid, creatinine, urea nitrogen (BUN), and XOD activities

The levels of uric acid, Cr, and BUN were determined by 7600 Automatic Biochemical Analyzer (Hitachi High‐Technologies). Hepatic and serum XOD activity were measured according to the manufacturer's instruction (Wuhan Huamei Bioengineering Co., Ltd.).

### 2.6 Histopathological examination

The rats were anesthetized with 10% chloral hydrate 1 h after the last intragastric administration. After the kidney tissue was removed, it was washed with normal saline, dried with filter paper, weighed, and placed in a 15 ml centrifuge tube. A 5% paraformaldehyde solution was added to the 15 ml centrifuge tube through a pipette, and kidney tissue was fixed for 24 h. The tissue was made into paraffin‐embedded slices, which were dehydrated, transparent, paraffin‐impregnated, and paraffin‐embedded. Paraffin‐embedded slices were sliced by rotary slicer, spread and baked at 50°C for 3 h, then dewaxed with xylene and gradient alcohol. Hematoxylin‐eosin staining (H&E stain) was used to stain this tissue. After dewaxing, washing, differentiation, blue refraction, eosin staining, dehydration, transparency, and sealing, the pathological changes of renal tissue were observed under a microscope, which was used to analyze the modeling and drug treatment of hyperuricemia in rats.

### 2.7 RNA isolation and real‐time PCR analysis

The kidneys were extracted for extraction of total RNA using TRIzol reagent. Next, cDNA was synthesized from the total RNA: 1 μg of total RNA was subjected to reverse transcription PCR using Superscript III First Strand (Invitrogen, USA), and the obtained cDNA was diluted in DNase‐free water Next, 2.0 μl of cDNA was amplified by real‐time PCR at the appropriate conditions. The primers used were as follows: URAT1, 5ʹ‐CTCTGCCTTTCTCCTGTTGA‐3ʹ and 5ʹ‐CCCCTTGATGATGACCTTG‐3ʹ; ABCG2, 5ʹ‐GGTTGTTGTAGGGCTCAC‐3ʹ and 5ʹ‐CCTCGGTATTCCATCTTT‐3ʹ; OATI, 5ʹ‐AGGGCTCTGTGACTCTGGTG‐3ʹ and 5ʹ‐CGAATCTTGCCTCCGCTTTA‐3ʹ; glucose transporter 9 (GLUT9), 5ʹ‐CAAAGACGAGGAAGCAGTAG‐3ʹ and 5ʹ‐TTCATTATCGCAGGCACA‐3ʹ; GAPDH, 5ʹ‐GACATGCCGCCTGGAGAAAC‐3ʹ and 5ʹ‐AGCCCAGGATGCCCTTTAGT‐3ʹ. GAPDH was used as an internal standard in the real‐time PCR.

### 2.8 Western blotting analysis

Renal tissues were homogenized in ice‐cold RIPA buffer supplemented with 1 mmol/L phenylmethylsulfonyl fluoride and then centrifugated at 12,000 rmp for 15 min. Protein concentration in the supernatant was determined using a bicinchoninic acid protein assay kit (Beyotime Biotechnology). Proteins were subjected to SDS‐polyacrylamide gel electrophoresis and then transferred onto PVDF membranes. Next, the PVDF membranes were blocked with 5% skim milk and incubated with the primary antibodies for URAT1, GLUT9, OAT1, ABCG2, and GAPDH at 40°C overnight (Table 1). Subsequently, the membranes were incubated with the appropriate secondary antibody at room temperature. The PVDF membrane was subsequently placed in an Odyssey infrared fluorescence scanning imaging system for scanning analysis to calculate the gray value. Each experiment was repeated three times with different samples.

**TABLE 1:** The antibodies used for Western blotting analysis

Manufacturer	Description	Catalog number
Thermo Fisher Scientific (Cambridge, MA, United States)	URAT 1 Antibody	PA5‐76559
Thermo Fisher Scientific	ABCG 2 Antibody	PA5‐78699
Abcam, Inc. (Cambridge, MA, United States)	OAT 1 Antibody	Ab135924
Thermo Fisher Scientific	GLUT 9 Antibody	PA5‐80026
Abcam, Inc.	GAPDH Antibody	Ab181602

### 2.9 Statistical analysis

All statistical analyses were performed using SPSS 20.0 (IBM Corp.,) and GraphPad Prism 5 (GraphPad Software) statistical software. All data are presented as the mean ± standard deviation (SD). Significant differences were assessed by one‐way analysis of variance (ANOVA) followed by Dunnett's *post hoc* test, and differences were considered statistically significant at *p *< 0.05.

## 3 RESULTS

### 3.1 Effects of Yi‐Suan‐Cha on serum uric acid, creatinine, and BUN levels in hyperuricemia rats

**FIGURE 1:**
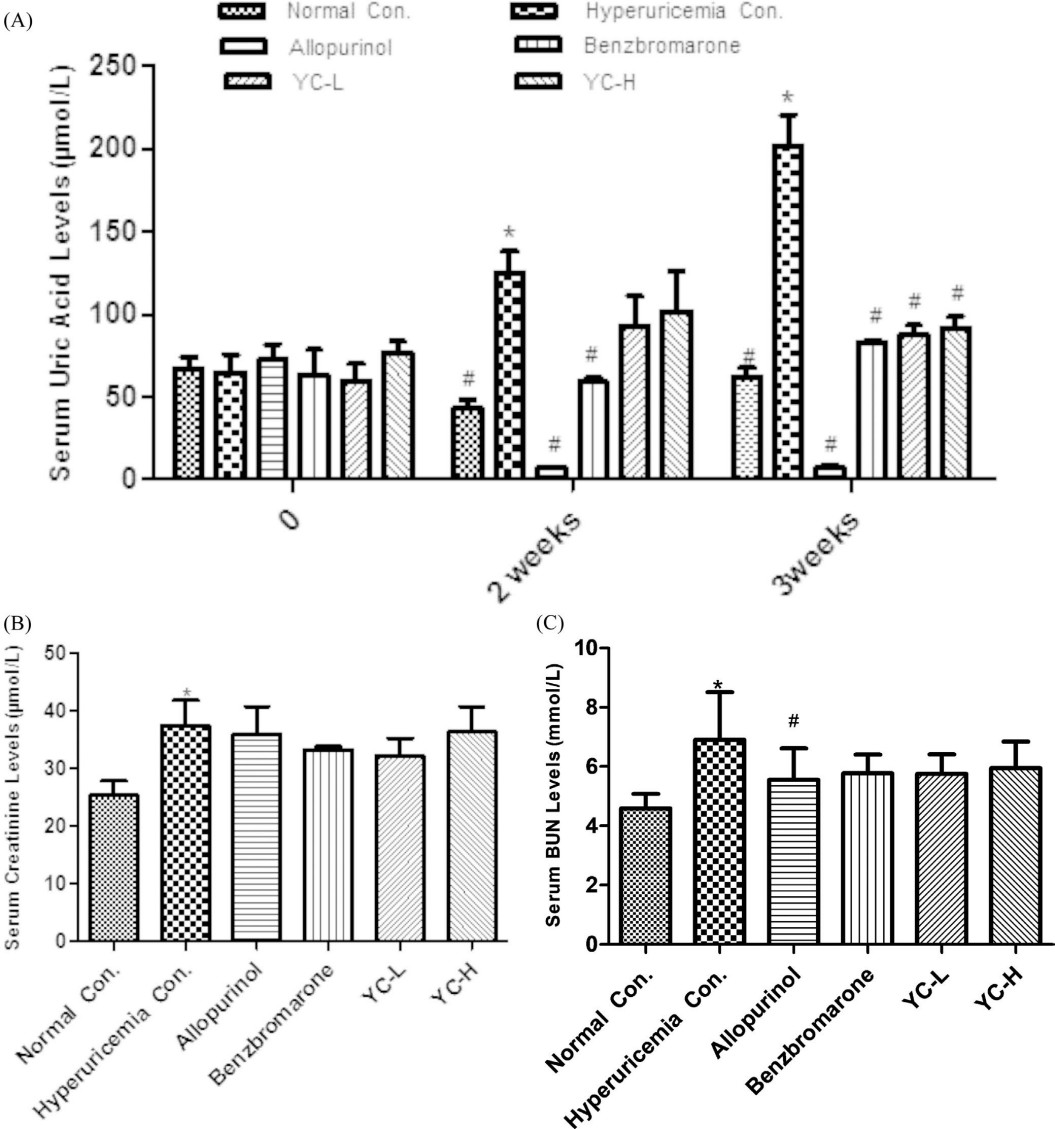
Effects of Yi‐Suan‐Cha on serum uric acid (A) and creatinine (B) and BUN (C) levels in rats. *p < 0.05 compared to the normal control; #*p* < 0.05 compared to the hyperuricemia control. Con., control; YC‐L, low‐dose Yi‐Suan‐Cha; YC‐H, high‐dose Yi‐Suan‐Cha

There was no significant difference in serum uric acid level between the groups before treatment (*p* > 0.05). After 3 weeks of treatment, compared with that in the normal control group (62.4 ± 11.30 μmol/L), serum uric acid increased significantly in the model group to 202.17 + 45.67 μmol/L, which suggested successful establishment of hyperuricemia model (*p* < 0.05), decreased in the allopurinol (7.50 ± 3.92 μmol/L, *p* < 0.05) group, and increased in the benzbromarone (83.33 ± 2.08 μmol/L, *p* < 0.05) groups. Importantly, both low‐dose and high‐dose Yi‐Suan‐Cha groups showed decreased serum uric acid level to 88.00 ± 11.22 and 91.80 ± 15.64 μmol/L (*p* < 0.05), respectively (Figure 1A). Additionally, Yi‐Suan‐Cha showed no significant effect on serum creatinine and BUN levels (Figure 1B–C).

### 3.2 Effects of Yi‐Suan‐Cha on histopathology of renal tissues in experimental rats

**FIGURE 2:**
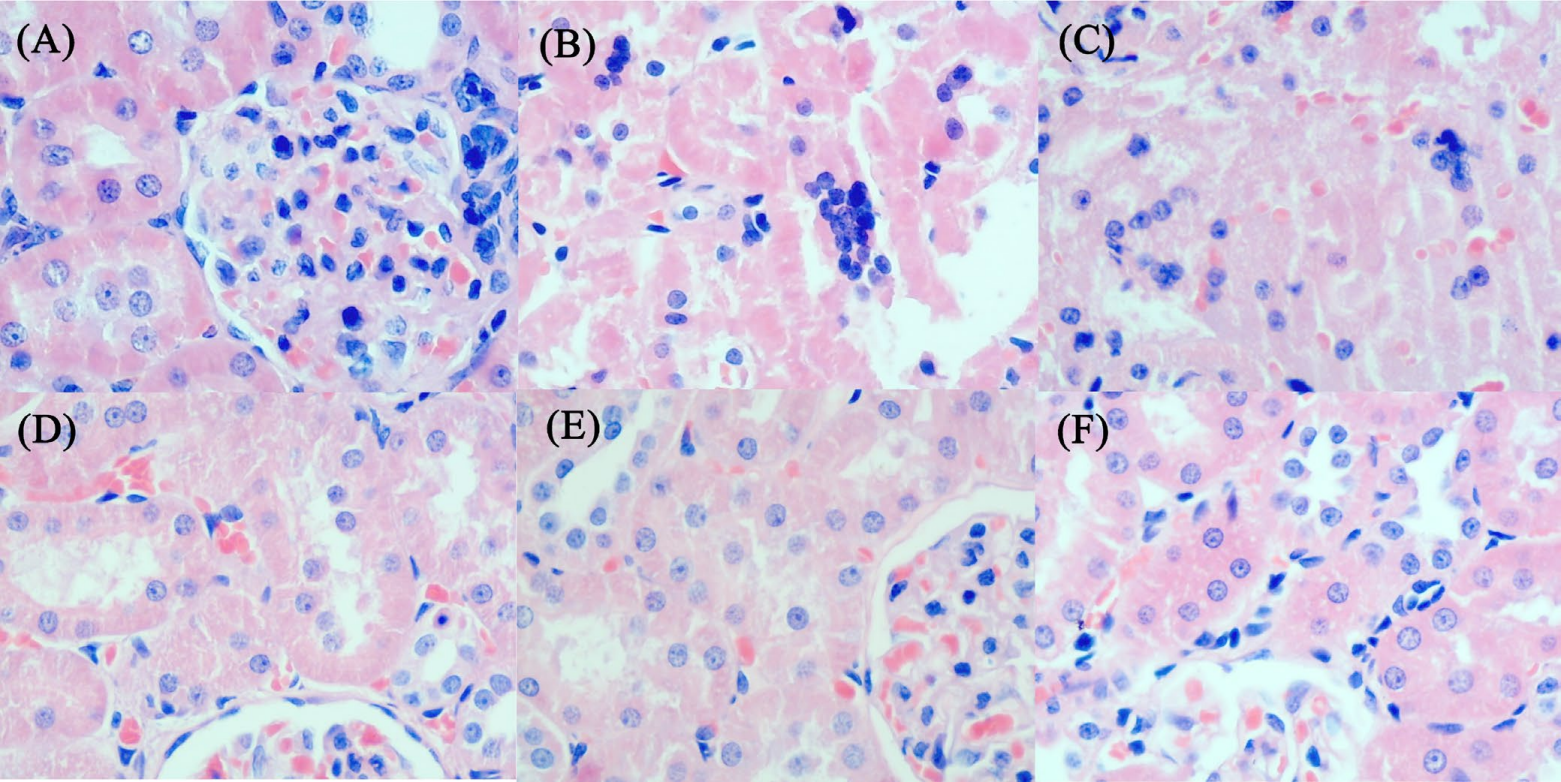
FIGURE Effect of Yi‐Suan‐Cha on renal tissue histopathology of hyperuricemia rats (hematoxylin‐eosin staining; magnification, ×400). (A) Normal control, (B) hyperuricemia control, (C) allopurinol, (D) benzbromarone, (E) low‐dose Yi‐Suan‐Cha, and (F) high‐dose Yi‐Suan‐Cha

The normal control group showed intact structure of glomeruli and tubules in the kidneys (Figure 2A). The model control and allopurinol groups showed shedding necrosis in the renal tubules (Figure 2B–C). The benzbromarone, low‐dose Yi‐Suan‐Cha, and high‐dose Yi‐Suan‐Cha groups showed clear kidney morphological structure with no obvious abnormalities (Figure 2D–F).

### 3.3 Effects of Yi‐Suan‐Cha on XOD expressions analysis in experimental rats

**FIGURE 3:**
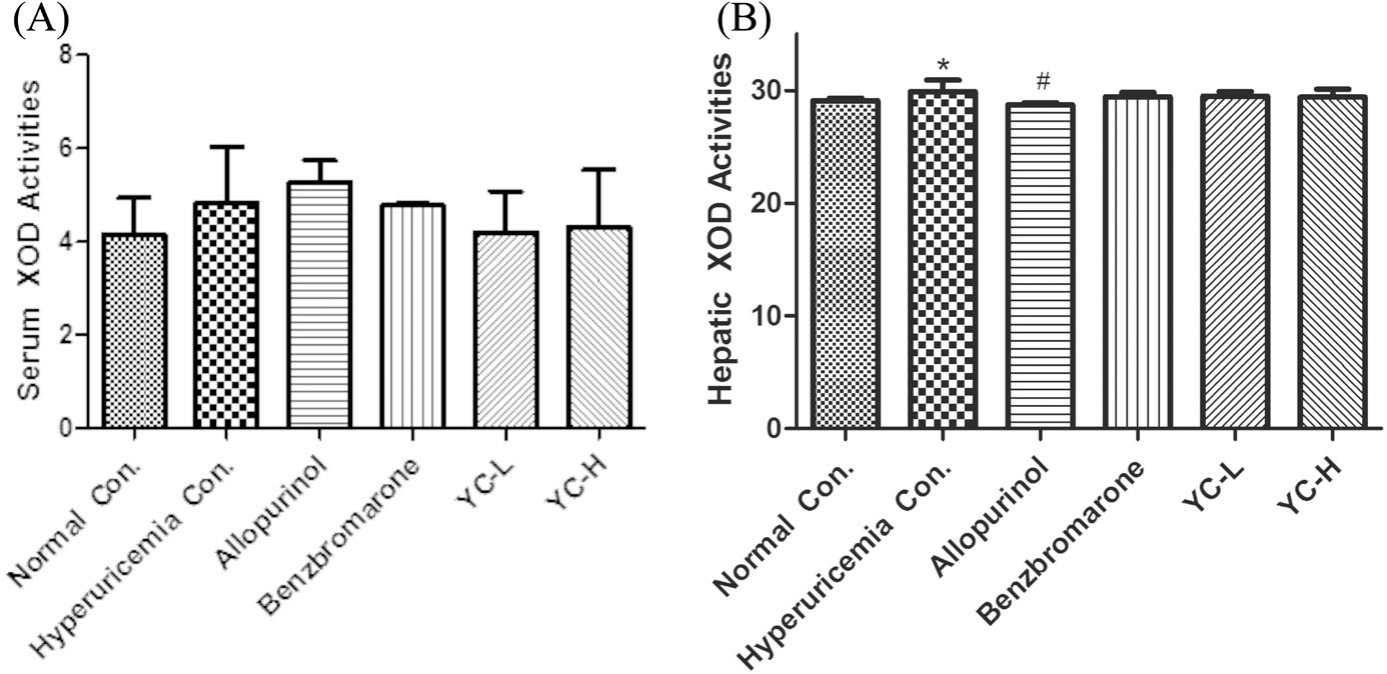
Effects of Yi‐Suan‐Cha on XOD activities in rat serum (A) and liver (B). *p < 0.05 compared to the normal control; #*p* < 0.05 compared to the hyperuricemia control. Con., control; YC‐L, low‐dose Yi‐Suan‐Cha; YC‐H, high‐dose Yi‐Suan‐Cha

Compared with the hyperuricemia group, the allopurinol group showed decreased hepatic XOD activity (*p* < 0.05, Figure 3), whereas the benzbromarone, low‐dose Yi‐Suan‐Cha, and high‐dose Yi‐Suan‐Cha groups did not show reduced serum and hepatic XOD activity.

### 3.4 Effects of Yi‐Suan‐Cha on the mRNA expression of renal transporters in rats

**FIGURE 4:**
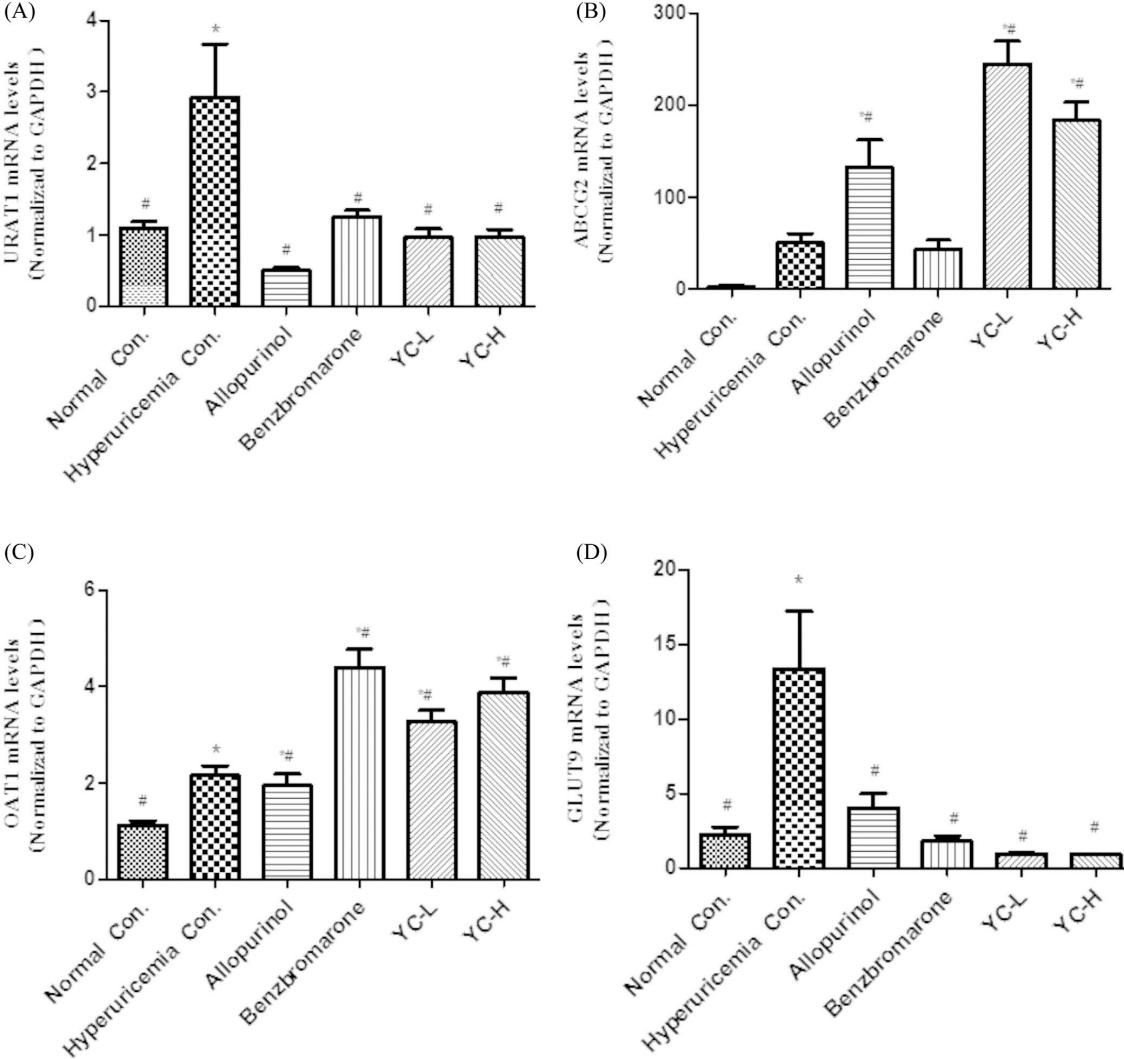
Effects of Yi‐Suan‐Cha on renal mRNA expression of (A) URAT1, (B) ABCG2, (C) OAT1, and (D) GLUT9. *p < 0.05 compared to the normal control; #*p* < 0.05 compared to the hyperuricemia control. Con., control; YC‐L, low‐dose Yi‐Suan‐Cha; YC‐H, high‐dose Yi‐Suan‐Cha

The effect of Yi‐Suan‐Cha on the mRNA expression of renal transporters is shown in Figure 4. Compared with that in the normal control group, the mRNA expression of URAT1 and GLUT9 in the model group was increased. Compared with the model group, the low‐dose and high‐dose Yi‐Suan‐Cha groups showed decreased mRNA expression of URAT1 and GLUT9 and increased mRNA expression of ABCG2 and OAT1 (*p* < 0.05, Figure 4A–D).

### 3.5 Effects of Yi‐Suan‐Cha on the protein expression of renal transporters in rats

**FIGURE 5:**
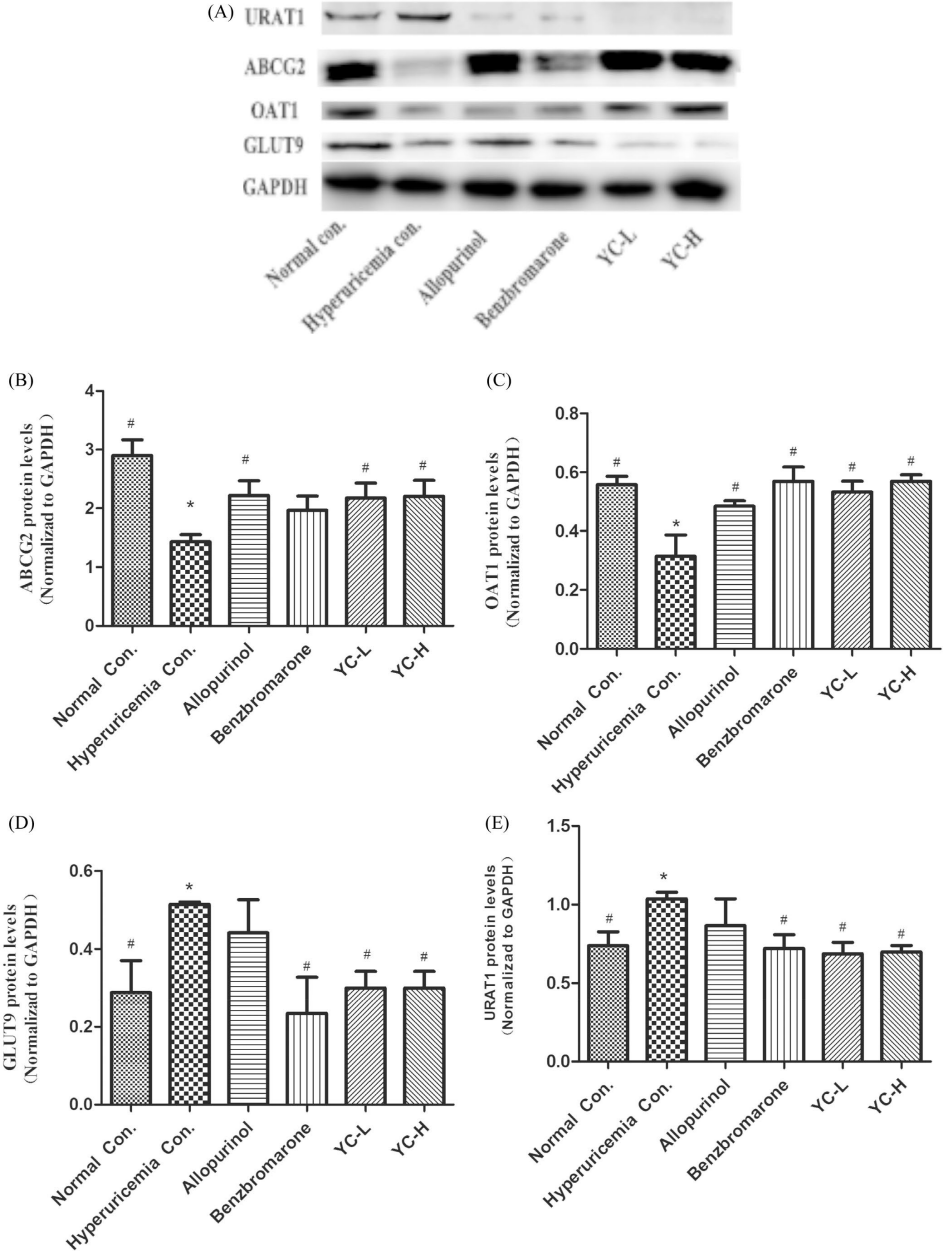
Effects of Yi‐Suan‐Cha on the protein levels of renal transporters. Proteins were extracted from the kidney for Western blot analysis of URAT1 (A, B), ABCG2 (A, C), OAT1 (A, D), and GLUT9 (A, E). *p < 0.05 compared to the normal control; #*p* < 0.05 compared to the hyperuricemia control. Con., control; YC‐L, low‐dose Yi‐Suan‐Cha; YC‐H, high‐dose Yi‐Suan‐Cha

The effect of Yi‐Suan‐Cha on the protein expression of URAT1, ABCG2, OAT1, and GLUT9 in the kidney was analyzed by Western blotting (Figure 5). Compared with the normal control group, the model group showed increased expression of URAT1 and GLUT9 proteins and decreased expression of ABCG2 and OAT1 proteins. Compared with the model group, the low‐dose and high‐dose Yi‐Suan‐Cha groups showed decreased expression of URAT1 and GLUT9 proteins and increased expression of ABCG2 and OAT1 proteins (*p* < 0.05, Figure 5A–E).

## 4 DISCUSSION

Studies have shown that hyperuricemia increases the risk of renal failure in healthy individuals and that lowering blood uric acid levels can delay CKD progression. Thus, maintenance of normal blood uric acid level is important to prevent the occurrence of kidney disease. Therefore, this is study is, to our knowledge, the first to investigate the urate level‐lowering effect and the associated mechanism of Yi‐Suan‐Cha in rats with hyperuricemia.

Potassium oxonate and adenine are commonly used to establish animal models of hyperuricemia. In this study, concomitant oral administration of PO and adenine‐induced hyperuricemia in rats, as confirmed by significant increases in serum uric acid levels in the model group.^23^ Our results showed that the serum uric acid levels in the model group were significantly greater after 3 weeks of treatment with PO and adenine than before treatment, indicating successful establishment of hyperuricemia model. Moreover, this result was supported by the histopathological findings of exfoliated and necrotized renal tubules in the hyperuricemia model group.

The mechanism of increased uric acid production is clear, and the defects of key enzymes in the process of sputum metabolism, that is, increased XOD activity, are the main cause of increased uric acid production. Thus, to elucidate the mechanisms underlying the anti‐hyperuricemia effect of Yi‐Suan‐Cha, we examined its effects on XOD activity. However, both low‐dose and high‐dose Yi‐Suan‐Cha could not reduce serum and hepatic XOD activity. Thus, Yi‐Suan‐Cha may have exerted its effects on the uric acid level through other mechanisms.

Genome‐wide association studies have shown that approximately 90% of hyperuricemia patients have insufficient uric acid excretion.^24^ The kidney and intestinal tract play a major role in uric acid excretion. More than 70% of uric acid excretion occurs through the kidney pathway.^25^ Uric acid is a polar molecule that does not pass freely through the cell membrane; therefore, it relies on ion channels to complete reabsorption and secretion in the renal proximal convoluted tubules. Thus, the key mechanisms of uric acid excretion are the renal tubular secretion and reabsorption processes. The renal uric acid transport process is regulated by various upstream and downstream expression of reabsorption and secretion transporters, which can be used as targets for reducing uric acid level.^26^ Various proteins have been identified to be involved in renal transport of uric acid. The most important transporters are the apical URAT1, basolateral GLUT9, and OAT1, which are directly related to uric acid homeostasis.^27^, ^28^, ^29^ URAT1 is encoded by the SLC22A12 gene and is mainly involved in reabsorption of uric acid in the renal proximal convoluted tubules. It is an important target of uric acid‐lowering drugs, such as fenofibrate, benzbromarone, and losartan.^30^ In this study, Yi‐Suan‐Cha downregulated the URAT1 protein possibly because the function of URAT1 is to reabsorb uric acid in the kidney; thus, Yi‐Suan‐Cha may enhance uric acid excretion though the downregulation of URAT1. GLUT9 is encoded by the SLC2A9 gene and involved in transport and reabsorption of urate. In this study, Yi‐Suan‐Cha promoted uric acid excretion by inhibiting reabsorption of uric acid by GLUT9. OAT1 is responsible for uric acid secretion.^31^ Notably, in this study, Yi‐Suan‐Cha showed dose‐dependent upregulation of OAT1 and downregulation of GLUT9 and URAT1. Taken together, these results provided evidence of the mechanism of the uric acid‐lowering effect of Yi‐Suan‐Cha in hyperuricemic rats.

Approximately a third of the uric acid content in the body is excreted via the intestine.^32^ ABCG2 plays an important part in this process. ABCG2 is a high‐volume exporter of uric acid that regulates uric acid excretion in the kidneys and intestines, as well as serum uric acid levels in humans.^33^ Overexpression of ABCG2 was observed in the intestine of nephrectomized rats, suggesting that the intestine plays a complementary role in uric acid excretion through ABCG2 in animals with poor renal function, such as those with end‐stage renal disease.^34^ Hosomi et al^35^ used potassium oxonate‐induced rat models of hyperuricemia to study intestine circulation and showed that the ABCG2 inhibitor acridine inhibits ABCG2‐mediated intestinal secretion of uric acid. Compared with wild‐type mice, ABCG2‐knockout mice show significantly higher serum uric acid level and significantly lower intestinal uric acid clearance rate, indicating that ABCG2 is the main transporter involved in intestinal elimination of uric acid.^36^ Yi‐Suan‐Cha may inhibit purine absorption in the intestine by upregulating the ABCG2 protein expression. Therefore, upregulating the ABCG2 protein expression may be related to the uric acid‐lowering effect of Yi‐Suan‐Cha. Considering that Yi‐Suan‐Cha possesses good pharmacological activity, screening of the bioactive compounds of Yi‐Suan‐Cha needs to be conducted in further studies for development of Yi‐Suan‐Cha as a treatment of hyperuricemia.

## 5 CONCLUSIONS

In conclusion, our study showed that Yi‐Suan‐Cha significantly reduced serum uric acid level in hyperuricemia rats, possibly by downregulating the expression of URAT1 and GLUT9 and upregulating the expression of ABCG2 and OAT1. Therefore, Yi‐Suan‐Cha showed high potential as an anti‐hyperuricemia drug.

## CONSENT FOR PUBLICATION

All participants gave consent for direct quotes from their interviews to be published in this manuscript.

## CONFLICT OF INTEREST

The authors declare that they have no competing interests.

## AUTHOR CONTRIBUTIONS

Fa‐quan Lin and Yuan‐yuan Qin conceived the idea and designed the study protocol. Xuan Zhang contributed with provision of study material. Hui Tao, Yangyang Wu, Jie Yan, Jianjun Meng, and Lin Liao collected, assembled data, and interpreted the data. Yuan‐yuan Qin and Xuan Zhang performed statistical analysis, wrote the manuscript.

## ETHICAL APPROVAL

Ethical approval was received from Guangxi Medical University Ethics Committee (ref: 201904021).

## Data Availability

The supporting materials used in this study are contained within the article.
